# Effect of Counterions on the Soft Ionization Mass Spectra of Analytes with Multiple Permanent Charges

**DOI:** 10.1021/acs.analchem.3c05786

**Published:** 2024-04-26

**Authors:** Olga Kočková, Petr Kasal, Jan Zelený, Zuzana Walterová, Věra Vlčková, Jindřich Jindřich

**Affiliations:** † Department of Analytical Chemistry, Institute of Macromolecular Chemistry of the Czech Academy of Sciences, Heyrovského nám. 2, Prague 6 162 00, Czech Republic; ‡ Department of Organic Chemistry, Faculty of Science, Charles University, Hlavova 8, Prague 2 128 43, Czech Republic

## Abstract

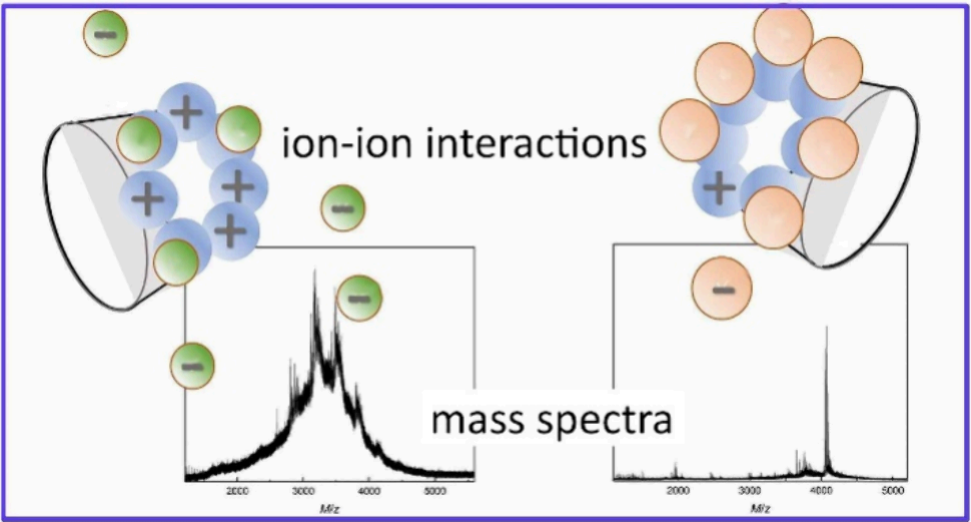

Multiply permanently charged analytes (MPCAs) are of great interest for various applications. MPCA soft ionization mass spectra (MS) strongly depend on the counterions of MPCA. We have studied thoroughly this effect to expand the use of MS in MPCA characterization. To this end, β-cyclodextrin-based MPCAs with 7 (MIM7NBCD) and 14 (MIM14BCD) quaternary ammonium charges with a series of monovalent counterions were prepared and their MS were measured using two of the most popular soft ionization techniques, electrospray ionization (ESI) and matrix-assisted laser desorption ionization (MALDI). MALDI MS of both analytes were well resolved, with signals assignable to the analytes only with the two least basic tested counterions (ClO_4_^–^ and TfO^–^). Similarly, analyte-assignable signals were observed in ESI MS of MIM14BCD only with ClO_4_^–^ and TfO^–^. The situation was opposite with ESI MS of MIM7NBCD where assignable signals were observed with Cl^–^ but not with TfO^–^. Thus, to get high-quality MS, binding between the MPCA permanent charges and the counterions must be of the optimal effective strength, given also by the number of analyte permanent charges as shown by the simple combinatorial model of binding. Of practical interest is the observation that unsuitable counterions can be replaced *in situ* by an excess of corresponding acid. The findings form a coherent framework for interpreting and improving MPCA mass spectra.

## Introduction

Mass spectrometry (MS) is one of the key methods of verifying the structure of synthesized compounds. The introduction of soft ionization techniques has increased its application field. During the characterization of cyclodextrin compounds with multiple permanent charges by two widely used soft ionization mass spectrometry techniques, electrospray ionization (ESI) and matrix-assisted laser desorption ionization (MALDI), we found that the obtained mass spectra strongly differed for analytes with chloride or with trifluoromethanesulfonate (triflate, TfO^–^) counterions. Not only did the charge state differ, but in most cases, the spectra were of such poor quality that the assignment of peaks was not possible.

The effect of counterions on the mass spectra has been reported both for ESI and MALDI. The average charge state in the ESI spectra of selected proteins decreased with the selectivity of the counterions toward anion exchange resins.^1^

The ESI mass spectra of several proteins in the presence of sodium salt Na^+^A^–^ showed a tendency to lose the HA in the order of gas basicity (GB) of the corresponding anions.^2^ GB of counterions also plays a role in MALDI processes. Distributions of anion adducts are visible in the MALDI mass spectra of ubiquitin in the presence of low GB anions; no adducts were observed in the presence of anions with higher GB.^3^

The positive charges of the peptides and proteins are generated by the reversible addition of H^+^ to an amine group. The ammonium/anion ion pair may be destroyed not only by splitting but also by elimination of neutral HA acid. The latter route is not possible for permanent charges such as quaternary ammonium. Nevertheless, the effect of the counterion on the charge distribution was found also for the analytes with multiple permanent charges (MPCA), namely, diquaternary ammonium compounds.^4^

Since MPCA are of interest for applications such as capillary zone electrophoresis, catalysis, or ionic liquids,^5,6^ the effect of counterions on the most frequently used ESI and MALDI MS is deemed to be worth investigating. For that purpose, β-cyclodextrin derivatives with 14 and 7 quaternary ammonium permanent charges were prepared with various monovalent counterions. The results are reported and analyzed here.

## Experimental Section

### Reagents

All solvents and reagents for syntheses were obtained from common commercial sources (Merck, Germany; Fluorochem, UK; Penta Chemicals, Czech Republic) and used without further purification. β-Cyclodextrin was purchased from Waco Chemicals (Germany). Water (HPLC LC-MS grade, VWR Chemicals, USA) and 2,5-dihydroxybenzoic acid (Merck, Czech Republic) were used for mass spectrometric analysis without further purification.

### Synthesis

**Figure 1:**
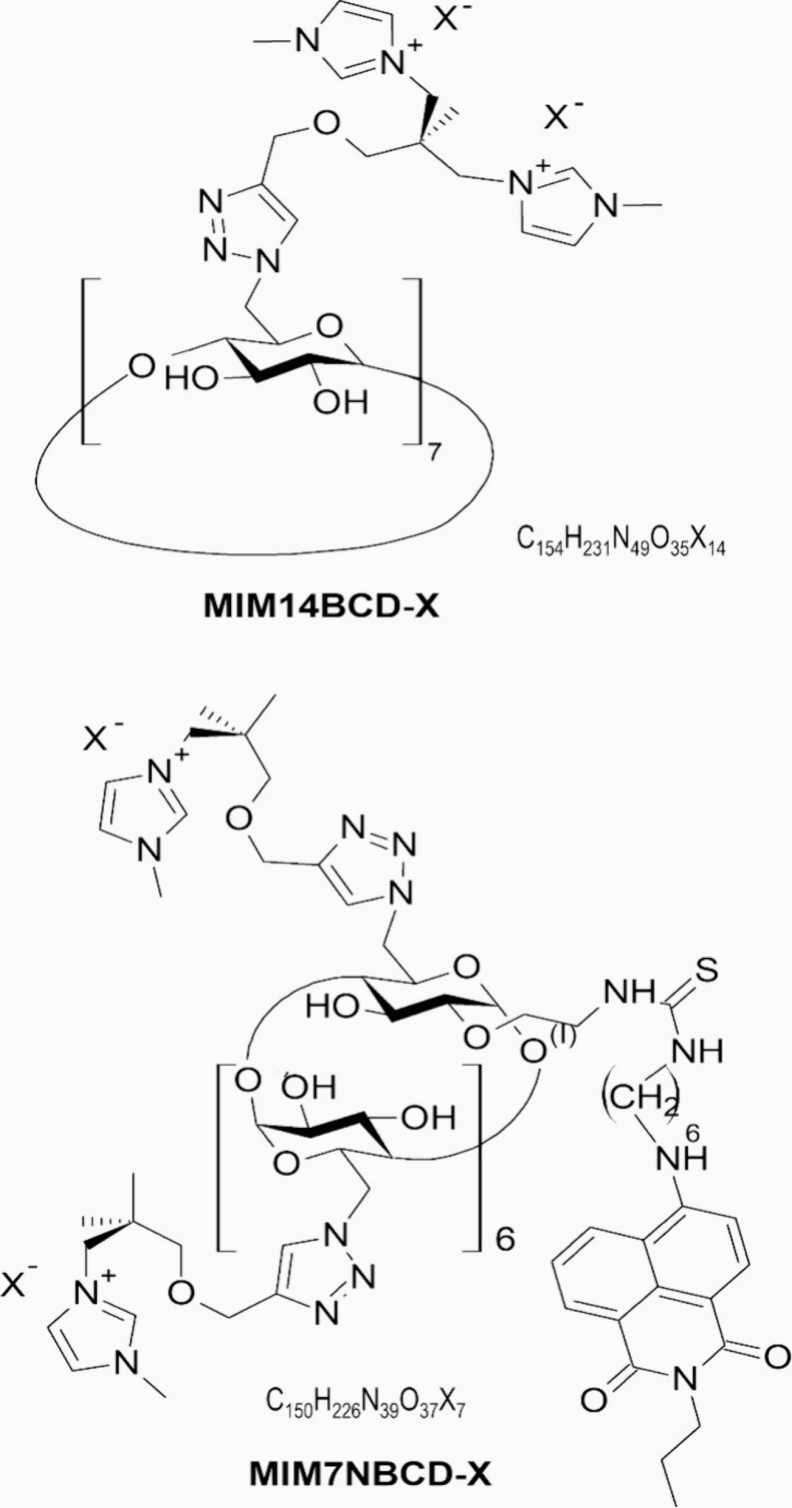
Structural and molecular formulas of MIM7NBCD-X and MIM14BCD-X, where X^–^ stands for the used anion.

Methylimidazolium β-cyclodextrin derivatives 6^I,II,III,IV,V,VI,VII^-heptadeoxy-6^I,II,III,IV,V,VI,VII^-heptakis[4-((2,2-dimethyl-3-(1-methyl-1*H*-imidazol-3-ium-3-yl)propoxy)methyl)-1*H*-1,2,3-triazol-1-yl]-2^I^-*O*-[2-(3-(6-((1,3-dioxo-2-propyl-2,3-dihydro-1*H*-benzo[*de*]isoquinolin-6-yl)amino)hexyl)thioureido)ethane-1-yl]-cyclomaltoheptaose hepta(hydrogen carbonate) and 6^I,II,III,IV,V,VI,VII^-heptadeoxy-6^I,II,III,IV,V,VI,VII^-heptakis[4-((2-methyl-3-(1-methyl-1*H*-imidazol-3-ium-3-yl)-2-((1-methyl-1*H*-imidazol-3-ium-3-yl)methyl)propoxy)methyl)-1*H*-1,2,3-triazol-1-yl]-cyclomaltoheptaose tetradeca(hydrogen carbonate) (MIM7NBCD-X and MIM14BCD-X; Figure 1) were prepared by published procedures (ref (7), compounds **95** and **91**). Bicarbonate counterions were subsequently replaced with selected counterions by adding stoichiometric amounts of matching acid aqueous solution to 2–5% solution of the corresponding bicarbonate in methanol or water; evaporation under reduced pressure followed. A general anion designation X can be replaced by a specific anion designation in the sample code. Characterization (NMR, IR, and HRMS) of the samples and their intermediates was given elsewhere.^7^

### ESI Mass Spectrometry

For electrospray mass spectrometry analyses, a mass spectrometer LCQ Fleet (Thermo Fisher Scientific, USA) was used. Solutions of samples (in water or in water and triflic acid) in a concentration of 0.1 mg/mL were introduced into the ESI source by continuous infusion with a 3 μL/min flow rate using an instrument syringe pump. Settings and conditions were as follows: spray voltage: 4.48 kV; capillary voltage: 36.98 V; tube lens voltage: 120 V; capillary temperature: 275 °C; sheath gas: nitrogen. The analyses were performed using positive-ion mode with an *m*/*z* range of 150*–*2000. The QuanBrowser program of the Xcalibur software was used for the evaluation.

### MALDI-ToF Mass Spectrometry

Mass spectra were obtained with an ultrafleXtreme ToF–ToF mass spectrometer from Bruker Daltonics, equipped with a 2000 Hz smartbeam-II laser (355 nm) using the positive-ion reflectron mode and panoramic pulsed ion extraction, after external calibration with poly(ethylene glycol).

Deposition of the samples onto the target plate was done by the dried droplet method.^8^ Water was used as a solvent both for the samples (10 mg/mL), and 2,5-dihydroxy benzoic acid (20 mg/mL) was used as a matrix. The mixing volume ratio sample:matrix was 1:5; the volume deposited on the target was 0.5–1 μL.

## Results and Discussion

**Figure 2:**
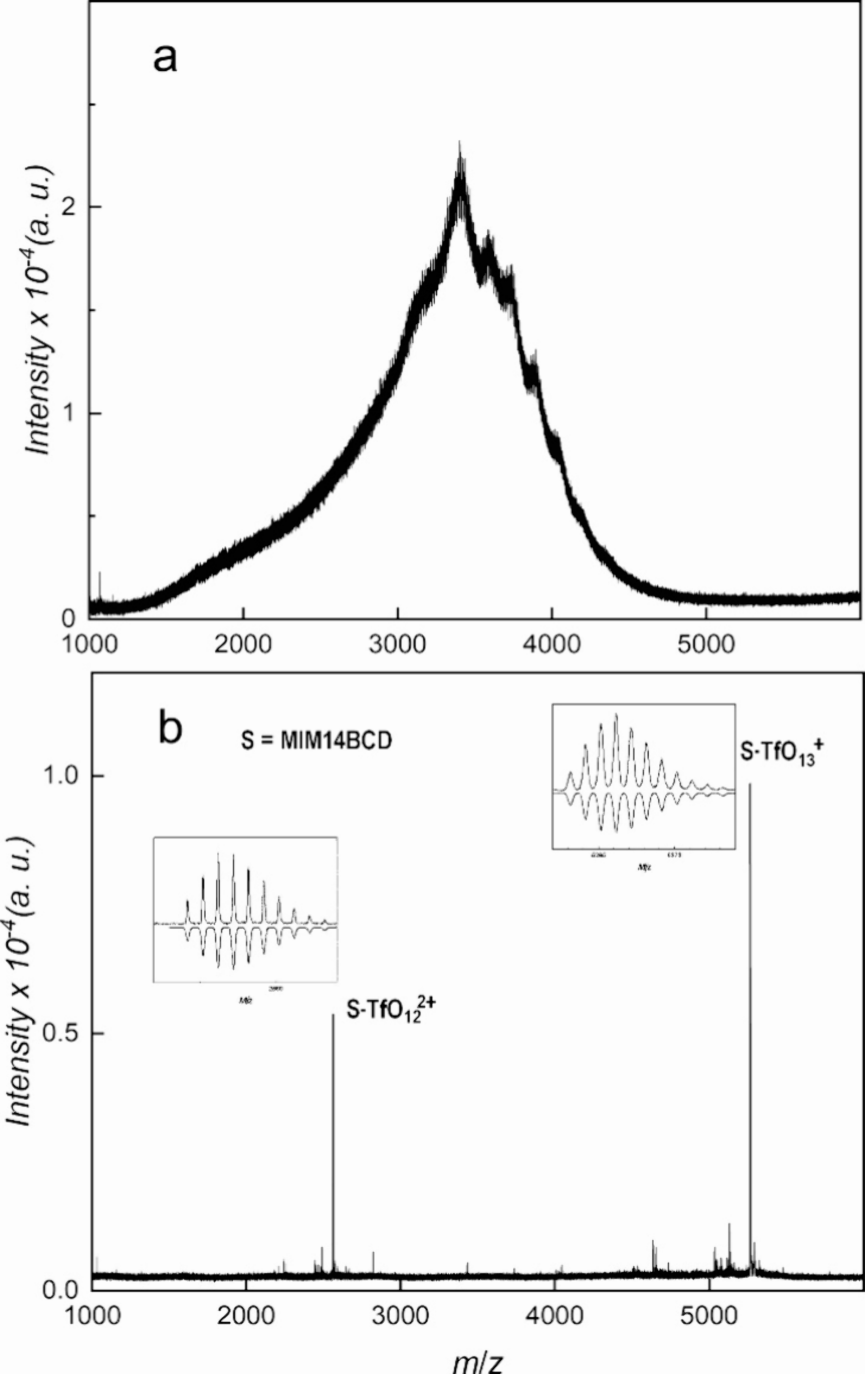
MALDI-ToF mass spectrum of sample MIM14BCD-X. (a) X = Cl; (b) X = TfO. The isotopic patterns of marked peaks are compared with simulation in insets.

The MALDI-ToF mass spectra of MIM14BCD-X, β-cyclodextrin-based MPCA with 14 permanent quaternary ammonium charges per molecule, are presented in Figure 2 for counterions Cl^–^ and TfO^–^.

The spectrum of MIM14BCD-Cl consists of one dominant wide peak, probably formed from several unresolved peaks, with a maximum of around *m*/*z* 3420. On the other hand, the spectrum of MIM14BCD-TfO is well resolved with dominant peaks corresponding to the assumed analyte with one or two TfO^–^ detached as is confirmed by the agreement of their isotopic patterns with the simulation (see insets in Figure 2b). Thus, MIM14BCD-TfO, despite having 14 permanent charges in the molecule, gives a MALDI-ToF mass spectrum with a standard number of free charges per molecule, while the remaining permanent charges are compensated by the original TfO^–^ counterions. The position of the wide peak in the spectrum of MIM14BCD-Cl indicates the dominance of species with one free charge, while the remaining permanent charges are not compensated by Cl^–^ (compare Table S1 with expected *m*/*z* values for MIM14BCD-X with various counterions and number of free charges). The width and low resolution of the peak can be attributed to in-source processes.^9^

**Figure 3:**
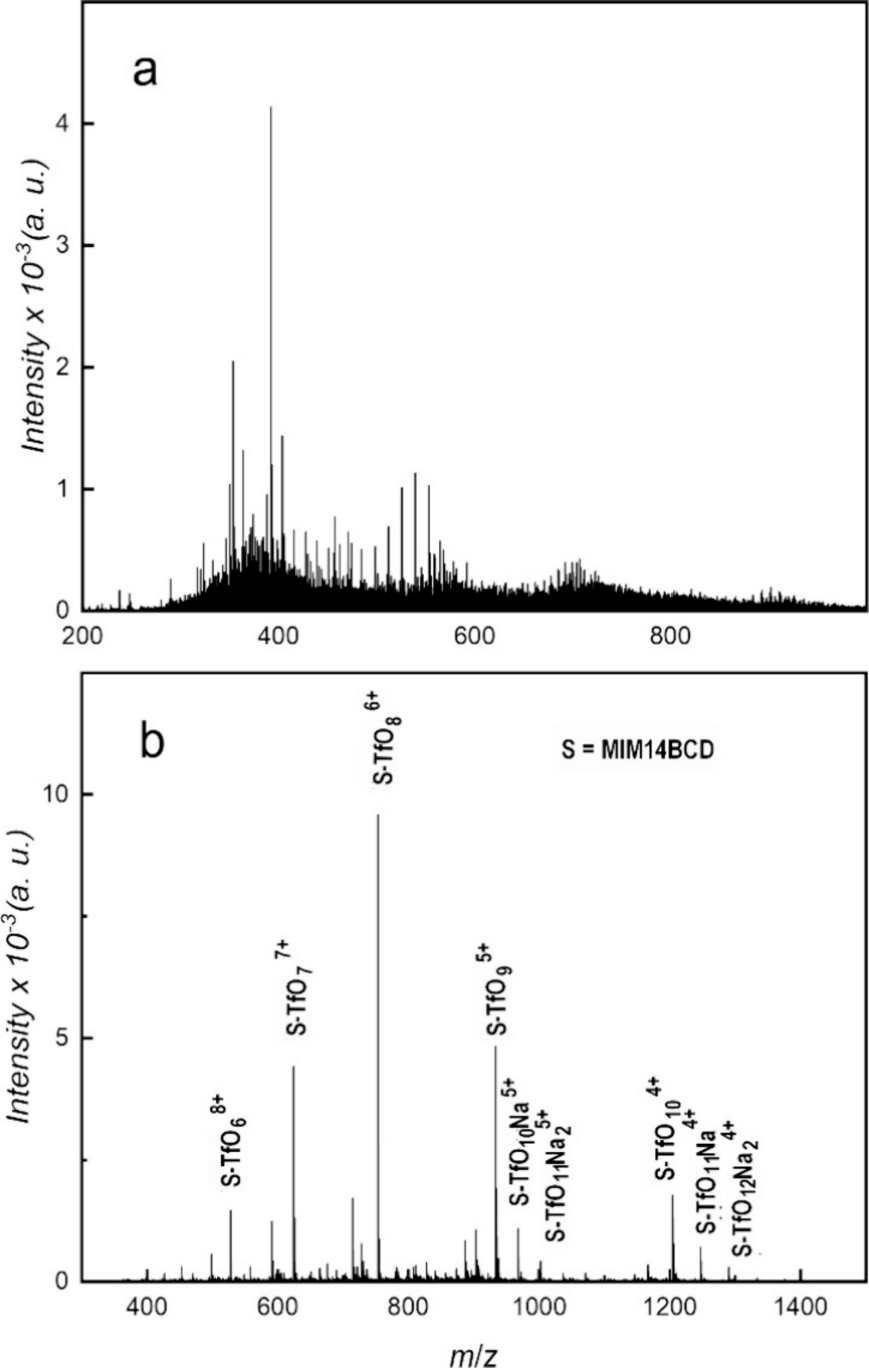
ESI mass spectrum of MIM14BCD-X. (a) X = Cl; (b) X = TfO. The identified peaks are labeled.

Due to the different ionization mechanisms employed, the ESI mass spectra often represent species with a higher number of free charges than the MALDI spectra. This is the case also for MIM14BCD-X (Figure 3). The spectrum of MIM14BCD-Cl consists of many peaks, the most intense ones with *m*/*z* 300–600, none of which could be assigned to the analyte. On the other hand, the spectrum with TfO^–^ counterions is simpler and presents peaks of MIM14BCD-TfO with four to eight free charges. The most intense of them are due to the loss of TfO^–^, but some of the minor ones bear also one or two Na^+^ cations. The adducts with Na^+^ rather than those with H^+^ are observed because sodium is a ubiquitous contaminant, and it is known that the good mass spectra of carbohydrates can be obtained by sodiation but not by protonation.^10^

Thus, both MALDI and ESI led to mass spectra being useful in the analysis of quaternary ammonium MPCA with TfO^–^ counterions but not with Cl^–^. In addition, the compensated permanent charges of the observed peaks are paired with TfO^–^. This indicates stronger ion pairing for TfO^–^ than for Cl^–^, although that should be expected for more basic Cl^–^ from purely electrostatic consideration. At the same time, however, the more basic anion can more easily combine with other cations present, such as H^+^, and form a neutral molecule, which can be more easily removed from the vicinity of the permanent charge. The environment for such secondary reactions might be different for MALDI, a plume of desorbed matrix and analyte and other ionic species,^9^ and for ESI, a shrinking liquid droplet.^10,11^ Thus, different types of basicity may be relevant—gas basicity for MALDI and basicity in the used solvent for ESI. Various types of specific interactions between permanent charges and counterions^12,13^ may also play a role. To better assess the influence of the properties of the counterions on MPCA mass spectra, it is advisable to extend the set of studied counterions.

### Effect of Selected Counterions

Samples of MIM14BCD-X with a series of additional counterions—CH_3_COO^–^, HCO_3_^–^, CF_3_COO^–^, NO_3_^–^, and ClO_4_^–^—were prepared, and their MALDI and ESI mass spectra were measured (see Figures S1–S10). The results together with those for Cl^–^ and TfO^–^ are summarized in Table 1 with the anions listed in order of the Hofmeister series, from kosmotropic to chaotropic.^13^ The series correlates well with the basicities of anions in gas and also in water; those are given as p*K*_a_ of conjugate acids, and only p*K*_a_ of Cl^–^ seems to be significantly out of order. The change in the shape of the MALDI mass spectra is not gradual. The spectra can be divided into two groups: (i) those of MIM14BCD-X with the least basic anions ClO_4_^–^ and TfO^–^, which are well resolved with dominant signals of the given analyte with one or two counterions detached; (ii) the spectra for the remaining used anions, which are dominated by a wide unresolved peak. The maximum of the peak for all counterions is located in the same region around *m*/*z* = 3420. This indicates that more basic counterions of this group are replaced during ionization (or before it) universally. Hydroxide OH^–^ comes to mind not only because it is readily available in an aqueous solution but also because a signal corresponding to the exchange of one ClO_4_^–^ with OH^–^ is observed in the spectrum of MIM14BCD-ClO_4_ (Figure S5). Nevertheless, the maxima of wide peaks are located at significantly lower values of *m*/*z* than would correspond to 13 positive charges compensated with OH^–^. The electron or hydride ion transfer in the plume^16^ seems to be an acceptable alternative process.

**Table 1:** Summary of the Mass Spectra of MIM14BCD-X with Selected Counterions

anion^a^	GB (kJ/mol)^b^	p*K*_a_^c^	MALDI^d^	ESI^d^
CH_3_CO_2_^–^	1429	4.8	W *S1*	F *S6*
HCO_3_^–^	1439	6.4	W *S2*	F *S7*
Cl^–^	1373	–5.9	W *2a*	F *3a*
CF_3_CO_2_^–^	1325	0.5	W *S3*	F *S8*
NO_3_^–^	1330	–1.5	W *S4*	F *S9*
TfO^–^	1251	–14.7	**1**,2 *2b*	4–8,**6***3b*
ClO_4_^–^	1200	–15.2	**1**,2 *S5*	3–4,**5***S10*

a Anions are in the order of the Hofmeister series (ref (13)).

b Gas basicity, taken from http://webbook.nist.gov/.

c p*K*_a_ of conjugate acids taken from refs (14) and (15).

d Notation: W, a wide unresolved peak; F, many unassigned peaks; numbers, *z* of observed analyte signals, strongest in bold; corresponding figure in italics.

The MALDI mass spectra usually present mostly singly charged ions. The explanation that initially formed multiply charged ions are neutralized by secondary reactions in the plume seems to apply to MPCA with strongly basic counterions but is less acceptable for those with weakly basic counterions because the ion pairing exclusively with the original counterions was observed for TfO^–^ and ClO_4_^–^. The probability that the freed permanent charge will be compensated with the original type of counterion is not very high. Thus, the key factor controlling the quality of the MPCA MALDI mass spectrum is the stability of the ion pairs.

Similarly, the effective stability of the ion pairs may be important in ESI where a counterion detached from MPCA may get permanently lost due to the droplet fission during evaporation.^9,11^ Accordingly, the ESI spectra of MIM14BCD-X with ClO_4_^–^ and TfO^–^ counterions are dominated by signals of the analyte with three to seven and four to eight detached counterions, respectively. The ESI spectrum of MIM14BCD-ClO4 does not contain the signals of the Na adducts observed in the ESI spectrum of MIM14BCD-TfO. The reason may be the different histories of the samples leading to different Na contamination. On the other hand, the ESI spectrum of MIM14BCD-ClO4 (Figure S10) contains the signals of the adducts with OH^–^ observed with MALDI.

The ESI spectra of MIM14BCD-X with the more basic counterions of the second group also do not present a gradual change since all of them are similar to that of MIM14BCD-Cl, i.e., all of them consisted of a large number of signals, none of which could be straightforwardly assigned to the analyte.

### Effect of the Number of Permanent Charges

The probability of finding a dissociated ion pair on a molecule increases with the number of its permanent charges. Thus, the charge state and consequently the mass spectrum of MPCA would depend on the number of charges in the MPCA molecule. To verify that, β-cyclodextrin-based MPCA with seven permanent quaternary ammonium charges per molecule (MIM7NBCD-X = Cl or TfO) was prepared. To probe also the effect of transient charges, the MIM7NBCD-X molecule was modified by a single substituent bearing a secondary amine, prone to accept a proton (see Figure 1).

**Figure 4:**
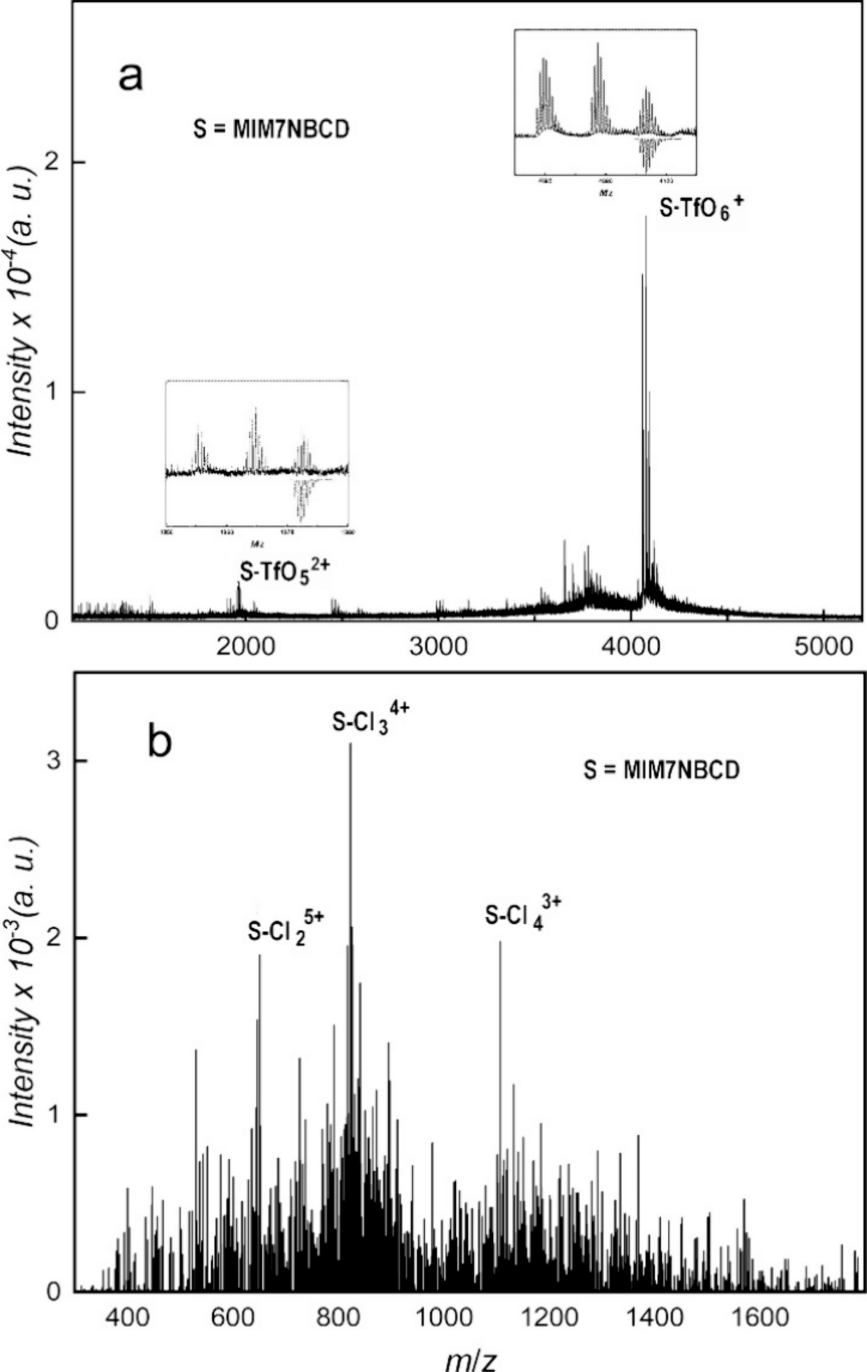
(a) MALDI mass spectrum of MIM7NBCD-TfO. The comparison with simulation is given in insets. (b) ESI mass spectrum of MIM7NBCD-Cl. The identified peaks are labeled.

The differences between the MALDI spectra of MIM7NBCD-Cl and MIM7NBCD-TfO are similar to those observed for MIM14CD-X, i.e., the spectrum of MIM7NBCD-Cl is of low resolution with a wide peak (Figure S11), whereas that of MIM7NBCD-TfO is of high resolution with signals corresponding to the one and two charges (Figure 4a). There is no evidence of ionization of secondary amine in the spectrum of MIM7NBCD-TfO; however, the peaks with *m*/*z* smaller by 16 and 34 Da than the singly charged peak 1 and by 8 and 17 Da than the doubly charged peak 2 are more intense than the peaks with expected values of *m*/*z*, see insets in Figure 4a. The additional peaks are related to the additional substituent as their counterparts are not found with MIM14BCD-TfO. Namely, the reactions on the thiourea linker are probably responsible for the additional peaks. Hydrogen sulfide can be eliminated from thiourea (loss of 34 Da), for example, by oxidation, and the resulting carbodiimide can be hydrated (addition of 18 Da, i.e., the net loss of 16 Da).^17^ The choice of appropriate counterions thus enables a detailed characterization of MPCA.

Unlike MALDI, ESI gave opposite results for MIM7NBCD-X compared to MIM14CD-X. No signals could be assigned to the analyte in the spectrum of MIM7NBCD-TfO (Figure S12), whereas the dominant peaks can be assigned to the analyte with some Cl^–^ lost in the spectrum of MIM7NBCD-Cl (Figure 4b). As in the MALDI spectrum of MIM7NBCD-TfO, the signals of MIM7NBCD-Cl are preceded by those of the analyte with the thiourea linker reacted (Figure S13). Since the signals for MIM7NBCD-X with the reacted linker are found both in MALDI and ESI spectra, the reactions at the thiourea linker occur before ionization.

Thus, it was confirmed that the shape and quality of the MPCA mass spectra depend not only on the effective strength of ion pairing but also on the number of permanent charges in a molecule.

The net charge distribution depends on the number of permanent charges per molecule because the probability of finding a dissociated ion pair on a molecule increases with the number of its permanent charges. Due to the Coulombic interactions, the probability of ion pair dissociation decreases with the number of free charges per molecule.

Assuming a uniform probability *p* that the counterion will be released from a given ion pair of a neutral molecule bearing *n* permanent charges and using a combinatorial approach,^18^ a simple general relation for the signal intensity of the analyte with *n* permanent charges from which is *x* free, *I*_*x*_^*n*^, can be derived
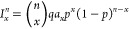
1where *q* accounts for the relation between the peak intensity and the fraction of corresponding analyte molecules. *a*_*x*_ is a factor accounting for the overall Coulombic anticooperativity of the release of *x* counterions. The values of *a*_*x*_ depend on *x* but may be assumed to be independent of the number of permanent charges for structurally similar analytes. Thus
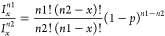
2

Equation 2 may be oversimplified; nevertheless, it helps to understand our results. The ratio calculated for *n*1 = 7 and *n*2 = 14 with 1 −*p* ≈ 1 (ion-to-neutral ratios is <10^–3^ for MALDI),^19^ given in Figure S14, strongly decreases with *x*. Each ionization method gives information about the analytes bearing a specific number of charges. The strength of ion pairing in MPCA should be optimal to achieve an optimal net charge distribution. The ion pairing of TfO^–^ and ClO_4_^–^ with quaternary ammonium is strong enough to generate predominantly singly charged adducts with the original counterions visible in MALDI spectra. Equation 2 explains why this is true both for MIM7NBCD-TfO and MIM14BCD-TfO because it predicts the peak intensity of a singly charged analyte with 7 permanent charges to be 50% of that of the analyte with 14 permanent charges, but at the same time, the peak intensity of an analyte with 7 permanent charges just 3% of that of the analyte with 14 permanent charges for analytes with four net charges. This explains why signals easily assignable to the analyte dominate the ESI mass spectrum of MIM14BCD-TfO, but none of such could be found in the spectrum of MIM7NBCD-TfO. Equation 2 also predicts that the ratio of peak intensity for *z* = 2–1 is lower for MPCA with 7 permanent charges than for MPCA with 14 permanent charges as was observed in the MALDI spectra of MIM7NBCD-TfO and MIM14BCD-TfO.

Quaternary ammonium ion pairs with Cl^–^ may be expected to be more easily separated during ionization than those with TfO^–^. Consequently, poor MALDI mass spectra are obtained for MIM7NBCD-Cl and MIM14BCD-Cl. Multiple free charges are compatible with ESI mass spectrometry if their numbers are within certain limits. Peaks corresponding to the analyte are detectable in the ESI mass spectrum of MIM7NBCD-Cl but not in that of MIM14BCD-Cl for which the number of free charges is probably too high because eq 2 predicts more free charges per molecule for an analyte with more permanent charges.

Equation 1, with *a*_*x*_ = 1, predicts that the number of free charges at which the highest signal intensity is achieved, *x*_max_, increases with decreasing strength of ion pairing, i.e., increasing *p*, and that *x*_max_ increases with the number of permanent charges, *n*. For very strong ion pairing, as may be expected to exist for TfO^–^ in MALDI, *x*_max_ = 1 will hold both for *n* = 7 and 14. Different effective strengths of ion pairing may be assumed for MALDI and ESI due to their different mechanisms. Weaker but still strong ion pairing of TfO^–^ in ESI will result in *x*_max_ = 1 for *n* = 7 leading to *m*/*z* above the detection limit of the method for MIM7NBCD-TfO but to the higher and detectable *x*_max_ for MIM14BCD-TfO.

Weak ion pairing of Cl^–^ leads to the loss of the original counterions and formation of multiple free charges per molecule, which are compensated by various secondary reactions in the plume during MALDI, which results in the deterioration of MALDI mass spectra both of MIM7NBCD-Cl and MIM14BCD-Cl. The effective strength of Cl^–^ ion pairing and the secondary reactions occurring during ESI would be different from that in MALDI. The experimental value of *x*_max_ = 4 was found for MIM7NBCD-Cl. Equation 1 predicts not only that *x*_max_ will be shifted to a higher value for MIM14BCD-Cl but also that the intensity of the corresponding signal will decrease. More importantly, the higher number of free charges increases the probability of secondary reactions confounding the ESI spectrum.

### Replacement of Counterions *In Situ*

**Figure 5:**
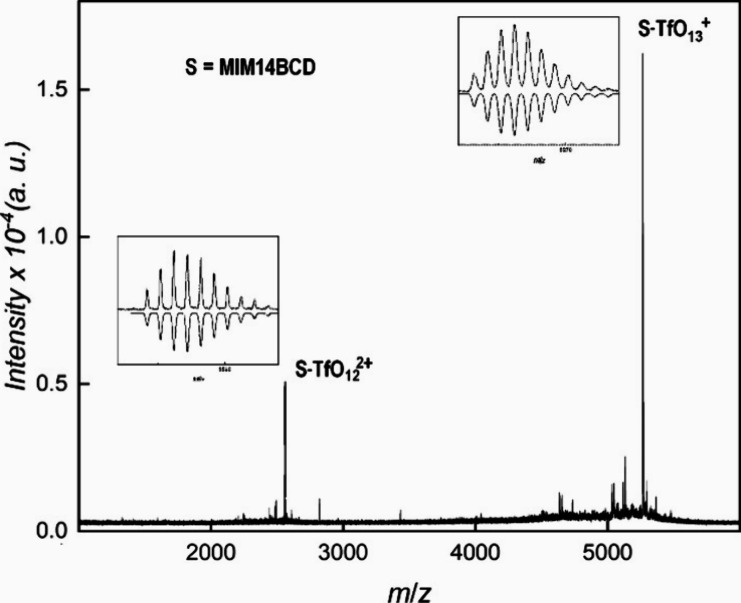
MALDI-ToF mass spectrum of sample MIM14BCD-Cl in the presence of TfOH in 10× molar excess over Cl^–^. The isotopic patterns are compared with simulations in insets.

The samples for which the MS structural confirmation will be required will usually carry counterions dictated by the intended application, and the exchange of counterions may be required to improve the mass spectra of MPCA. In the area of ionic liquids, such exchange is usually done by metathesis with silver salts.^20^ Since this would lead to significant contamination of the sample by silver ions, we tested the addition of triflic acid (TfOH) to the deposited solution for MALDI measurement. The excess triflate over chloride was necessary to improve the spectra of MIM14BCD-Cl. The spectrum of MIM14BCD-Cl with the addition of TfOH corresponded to that of MIM14BCD-Cl at a TfO^–^ to Cl^–^ molar ratio of 1:1 (Figure S15) and to that of MIM14BCD-TfO at a ratio of 10:1 (Figure 5).

The decision to replace counterions is not so clearly cut for ESI measurements since the quality of the spectrum also depends on other factors such as the number of permanent charges per molecule—the spectrum for more basic counterions can be better than that for TfO^–^. Nevertheless, if a poor ESI spectrum is obtained for more basic counterions, such as in the case of MIM14BCD-Cl, then adding TfOH to the sample solution before the injection is worth trying. As with MALDI, however, a high excess of TfOH has to be used to see the required effect. No assignable signals were found in the spectrum of MIM14BCD-Cl after the addition of TfOH at a TfO^–^ to Cl^–^ molar ratio of 1:1 (Figure S16). The spectrum quality significantly improved at a TfO^–^ to Cl^–^ molar ratio of 10:1, and the signals of adducts with 6–10 TfO^–^ counterions and no Cl^–^ were identified (Figure S17).

## Conclusions

The MALDI and ESI mass spectra of MPCA are frequently of low quality, with no apparent relation to the studied analyte. Changes in various experimental and instrumental parameters may be used in an attempt to optimize the spectra. In the present study, we concentrated on the two essential factors on which the quality of such spectra depends: the type of used counterion and the number of permanent charges per molecule of MPCA. The former depends on the nature of the analyte-charged groups and their counterions; the latter is given by the analyte structure. The studied anions can be divided into two groups according to the quality of the mass spectra of their salts with MIM14BCD-X, MPCA with 14 quaternary ammonium permanent charges. The high-resolution spectra were obtained for the most chaotropic and the least basic counterions. The remaining anions gave low-quality spectra, in which signals could not be assigned to the respective analytes. Thus, an effectively stronger ion pairing with quaternary ammonium can be assumed for anions of the first group. However, strong ion pairing can become counterproductive for MPCAs with lower numbers of permanent charges, which was not only observed for the ESI spectrum of MIM7NBCD-X but also explained by a simple combinatorial model. Only monovalent counterions were studied. The situation for the multivalent ones may be more involved due to cooperativity and the so-called parking effect.^18^ MPCAs with positive permanent charges, namely, quaternary ammonium, were studied. The derived simple combinatorial model used to interpret the results is independent of the sign of permanent charges, and thus, the conclusions based on it should be valid also for the mass spectra of MPCA with negative permanent charges. Nevertheless, caution should be exercised in applying the present findings to MS of such MPCA because the crucial factor, the effective strength of ion pairing, depends to a large extent on the secondary reactions, and these can vary with the permanent charge sign.

The quality and resolution of the MPCA spectra can be improved, and the analyte can be detected using a suitable counterion. MPCA counterions may be replaced by metathesis or acid–base reaction. We used the latter approach. With bicarbonate as the initial counterion, the challenging separation of the released byproducts^20^ was avoided. This will not be the case for other anions. The mass spectra of MPCA, however, can be improved *in situ* by the addition of the acid of suitable counterions in excess, here TfOH to replace Cl^–^. The findings, while not providing all of the details of the processes involved, form a coherent framework for interpreting and improving MPCA mass spectra.

## Supporting Information Available

Table of theoretical *m*/*z* values of MIM7NBCD-X and MIM14BCD-X adducts with various numbers of selected counterions; 16 additional mass spectra; graph of the effect of the number of permanent charges on the free charge distribution (PDF)
