# Supplementary material for: Effect of Counterions on the Soft Ionization Mass Spectra of Analytes with Multiple Permanent Charges
Source: Anal Chem. 2024 Apr 26;96(18):6940–6. doi: 10.1021/acs.analchem.3c05786 (PMC11079854; doi:10.1021/acs.analchem.3c05786)
Supplement: Supplementary file 1 — ac3c05786_si_001.pdf [file ac3c05786_si_001.pdf]

Supporting Information

Effect of Counter-Ions on Soft Ionization Mass Spectra of Analytes with Multiple Permanent Charges.

Olga Kočková[a]*, Petr Kasal[b], Jan Zelený[b], Zuzana Walterová[a], Věra Vlčková[a] and Jindřich Jindřich[b]

[a]Department of Analytical Chemistry, Institute of Macromolecular Chemistry of the Czech Academy of Sciences, Heyrovského nám. 2, Prague 6 162 00, Czech Republic

[b] Department of Organic Chemistry, Faculty of Science, Charles University, Hlavova 8, 128 43 Prague 2, Czech Republic

[*] Email: kockova@imc.cas.cz

Table of Content

Table of theoretical *m*/*z* values of MIM7NBCD-X and MIM14BCD-X adducts with various numbers selected counter-ions (Table S1)

16 additional mass spectra of MIM7NBCD-X and MIM14BCD-X (Figures S1-S13 & S15-S17)

Graph of the effect of the number of permanent charges on the free charge distribution. (Figure S14)

Table S1 Theoretical values of *m*/*z* for MIM14BCD-X and MIM7NBCD-X adducts with various numbers selected counter-ions

	MIM14BCD-X								MIM7NBCD-X		
X=	CH3CO2	HCO3	Cl	CF3CO2	NO3	ClO4	TfO	OH	Cl	TfO	OH
	Monoisotopic Mass										
$+H^+$	4153.97	4181.68	3817.35	4909.57	4195.61	4713.06	5413.11	3565.82	3443.46	4241.34	3317.69
$-X^-$	4093.95	4119.68	3781.38	4795.59	4132.62	4613.11	5263.16	3547.82	3407.49	4091.39	3299.69
$-2 X^-$	2017.47	2029.35	1873.20	2341.30	2035.32	2257.08	2557.10	1765.41	1686.26	1971.22	1641.34
$-3 X^-$	1325.31	1332.57	1237.15	1523.21	1336.22	1471.74	1655.08	1171.27	1112.52	1264.49	1088.56
$-4 X^-$	979.23	984.18	919.12	1114.16	986.66	1079.07	1204.08	874.20	825.64	911.13	812.17
$-5 X^-$	771.58	775.14	728.30	868.73	776.93	843.46	933.47	695.96	653.52	699.12	646.34
$-6 X^-$	633.15	635.79	601.09	705.11	637.11	686.39	753.07	577.13	538.77	557.77	535.78
$-7 X^-$	534.27	536.25	510.22	588.24	537.24	574.20	624.21	492.26	456.81	456.81	456.81
$-8 X^-$	460.11	461.59	442.07	500.59	462.34	490.06	527.56	428.60			
	Average Mass										
$+H^+$	4156.12	4183.66	3825.98	4911.72	4197.49	4721.65	5416.41	3567.67	4243.93	3448.72	3319.56
$-X^-$	4096.09	4121.66	3789.53	4797.71	4134.50	4621.22	5266.35	3549.67	4093.87	3412.27	3301.56
$-2 X^-$	2018.53	2030.33	1877.04	2342.35	2036.26	2260.90	2558.65	1766.33	1972.41	1688.41	1642.28
$-3 X^-$	1326.01	1333.22	1239.54	1523.90	1336.84	1474.12	1656.08	1171.89	1265.26	1113.79	1089.19
$-4 X^-$	979.75	984.66	920.79	1114.67	987.13	1080.73	1204.80	874.67	911.68	826.48	812.64
$-5 X^-$	771.99	775.53	729.54	869.14	777.31	844.70	934.03	696.33	699.53	654.09	646.71
$-6 X^-$	633.49	636.11	602.04	705.45	637.43	687.35	753.51	577.44	558.10	539.17	536.09
$-7 X^-$	534.55	536.52	510.97	588.53	537.51	574.95	624.58	492.52	457.08	457.08	457.08
$-8 X^-$	460.36	461.83	442.67	500.83	462.57	490.65	527.87	428.83			

[1)] X^- – counter-ion type

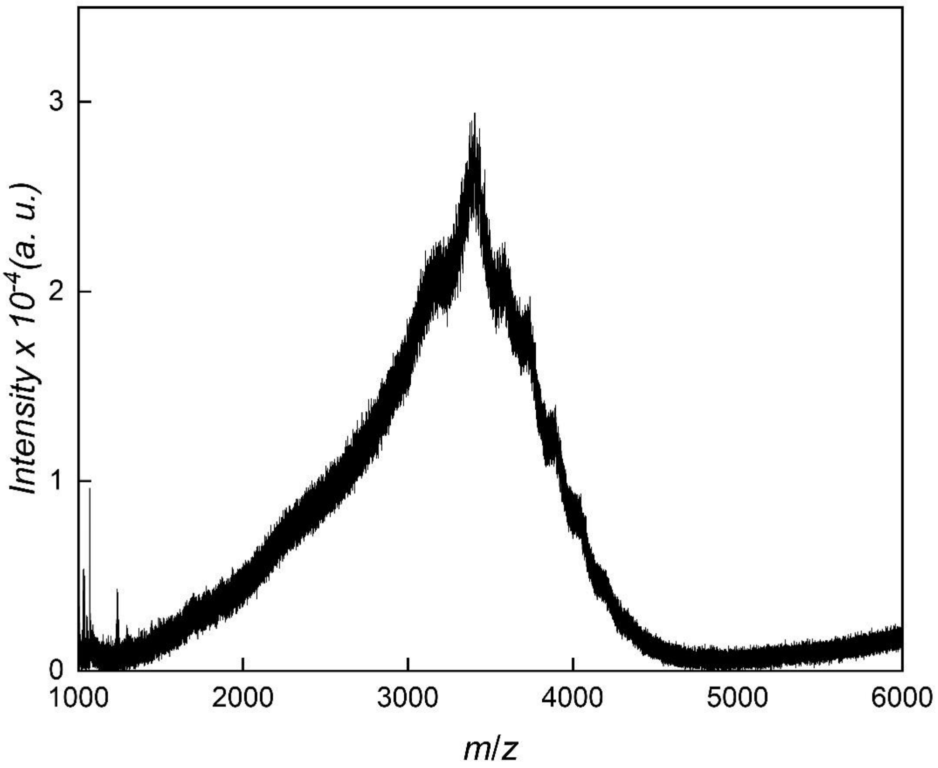

Figure S1. MALDI-TOF mass spectrum of the sample MIM14BCD-CH3CO2.

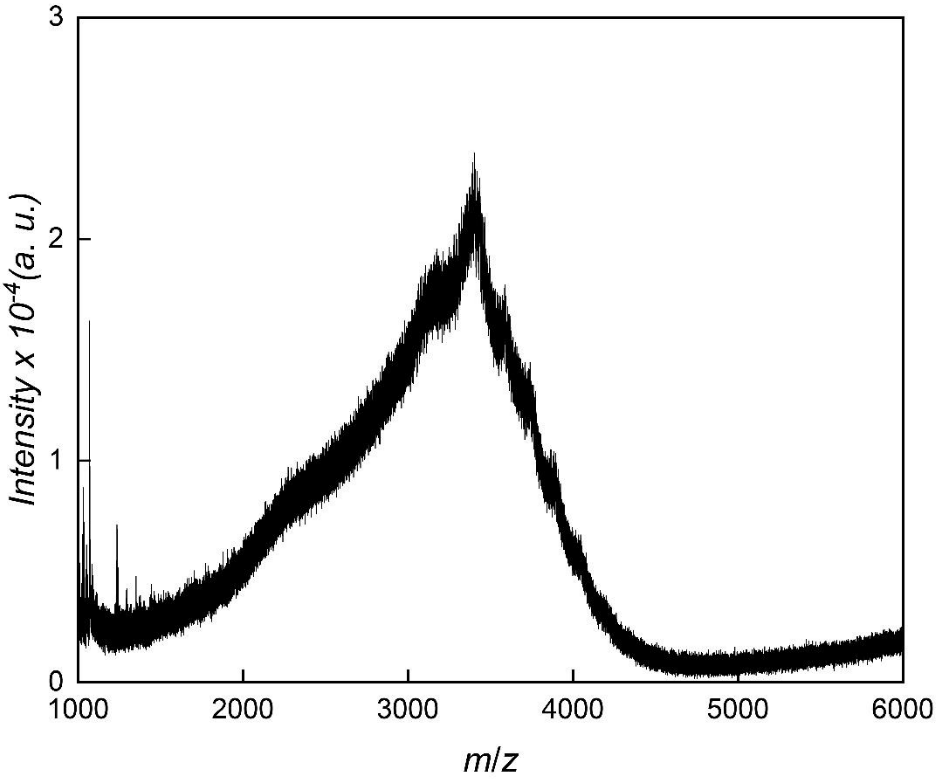

Figure S2. MALDI-TOF mass spectrum of the sample MIM14BCD-HCO3.

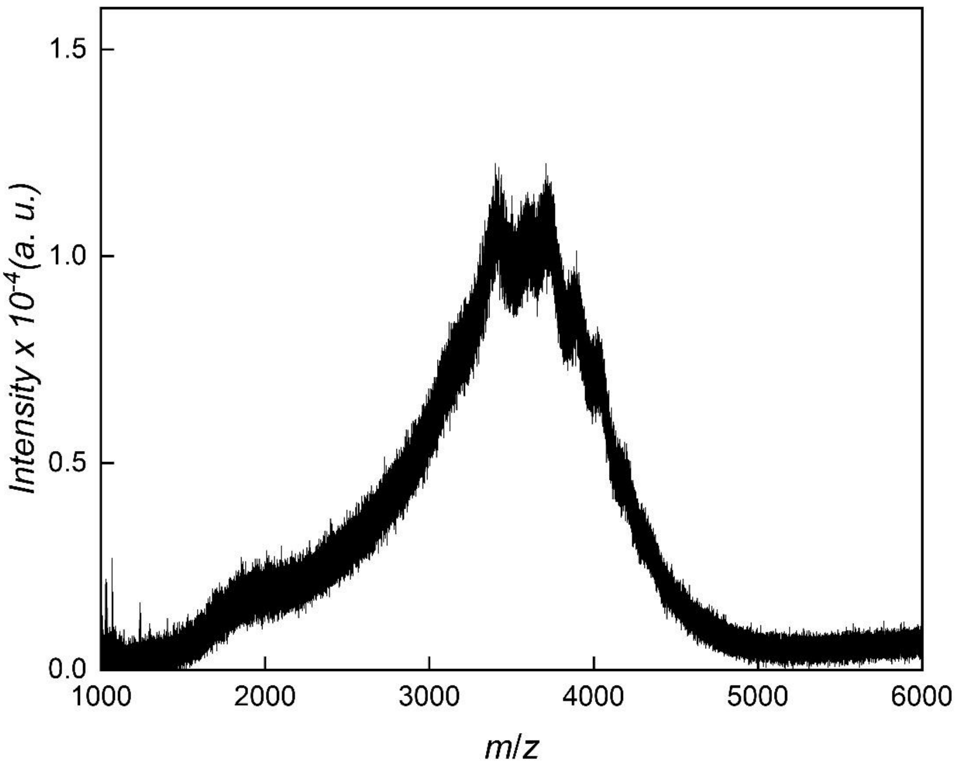

Figure S3. MALDI-TOF mass spectrum of the sample MIM14BCD-CF3CO2.

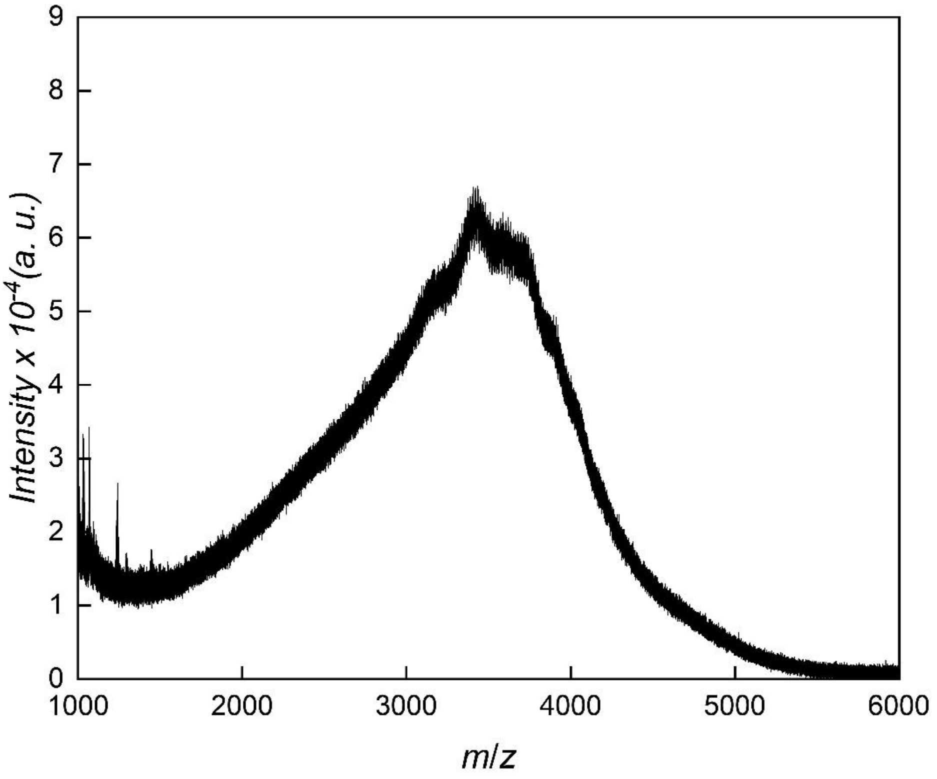

Figure S4. MALDI-TOF mass spectrum of the sample MIM14BCD-NO3.

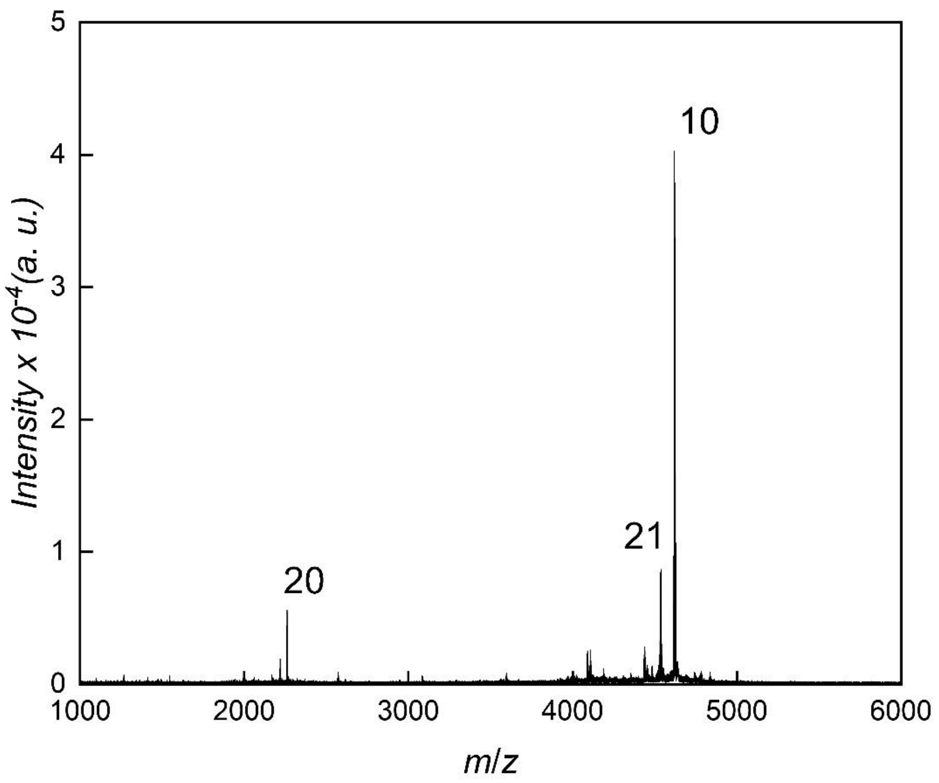

Figure S5. MALDI-TOF mass spectrum of the sample MIM14BCD-ClO4. The first digit of the peaks' labels corresponds to the number of detached CLO_4^- and the second one to the number of attached OH^-.

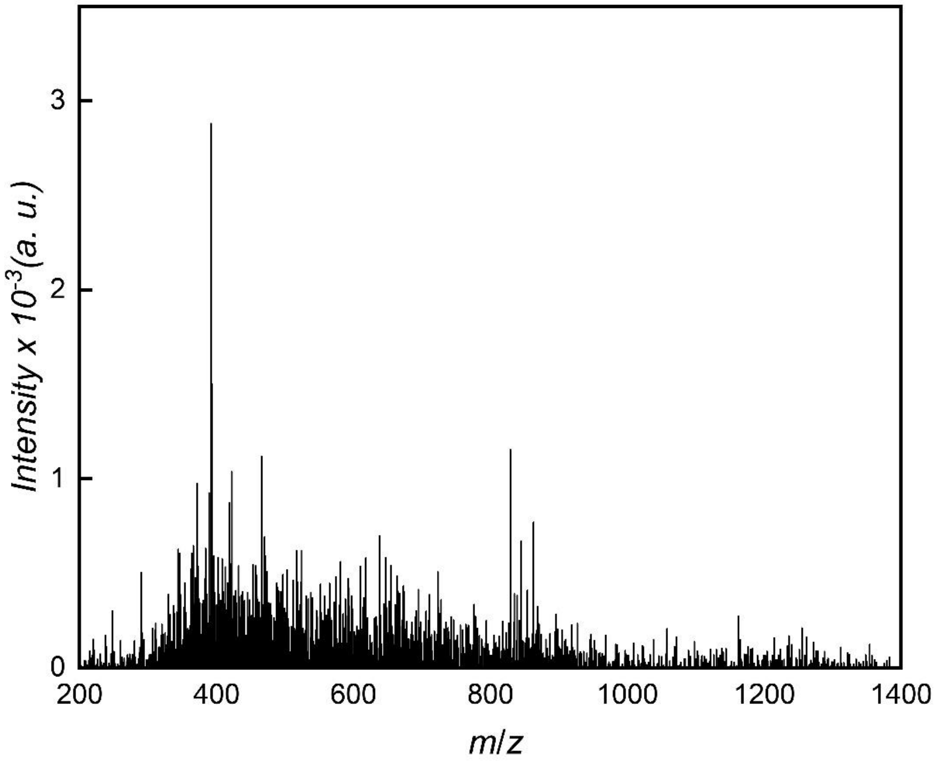

Figure S6. ESI mass spectrum of the sample MIM14BCD-CH3CO2.

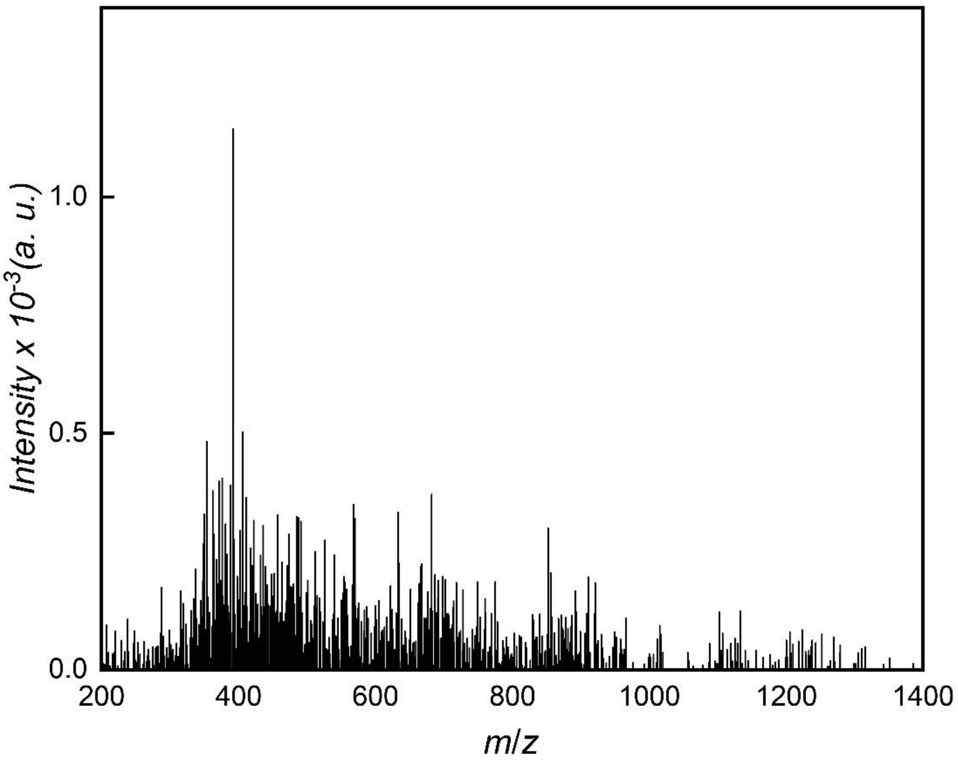

Figure S7. ESI mass spectrum of the sample MIM14BCD-HCO3.

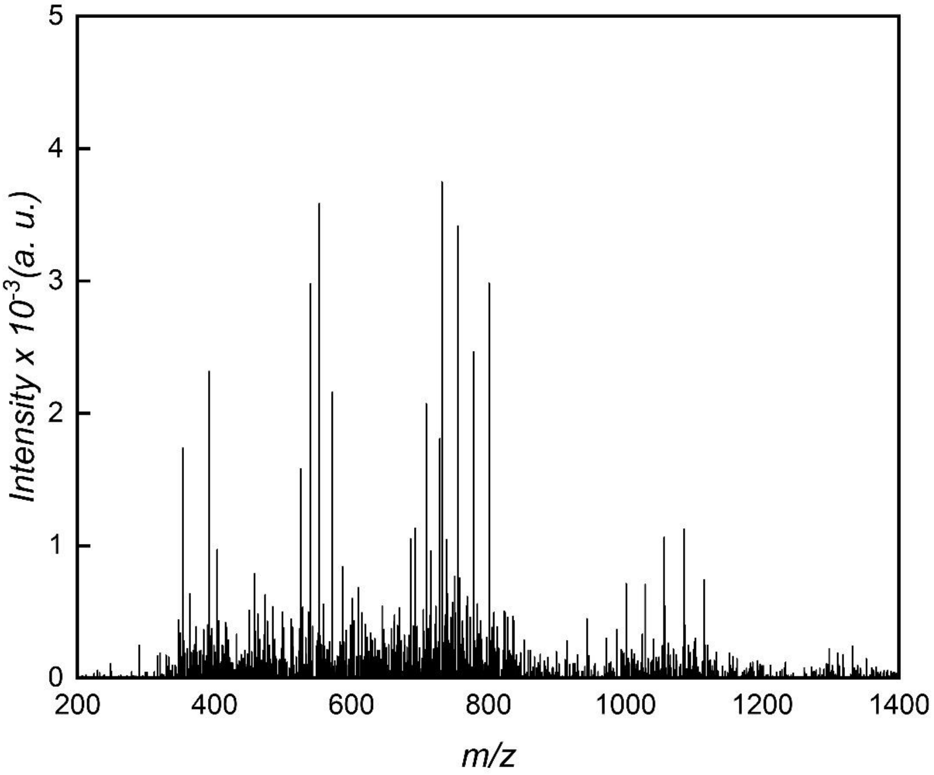

Figure S8. ESI mass spectrum of the sample MIM14BCD-CF3CO2.

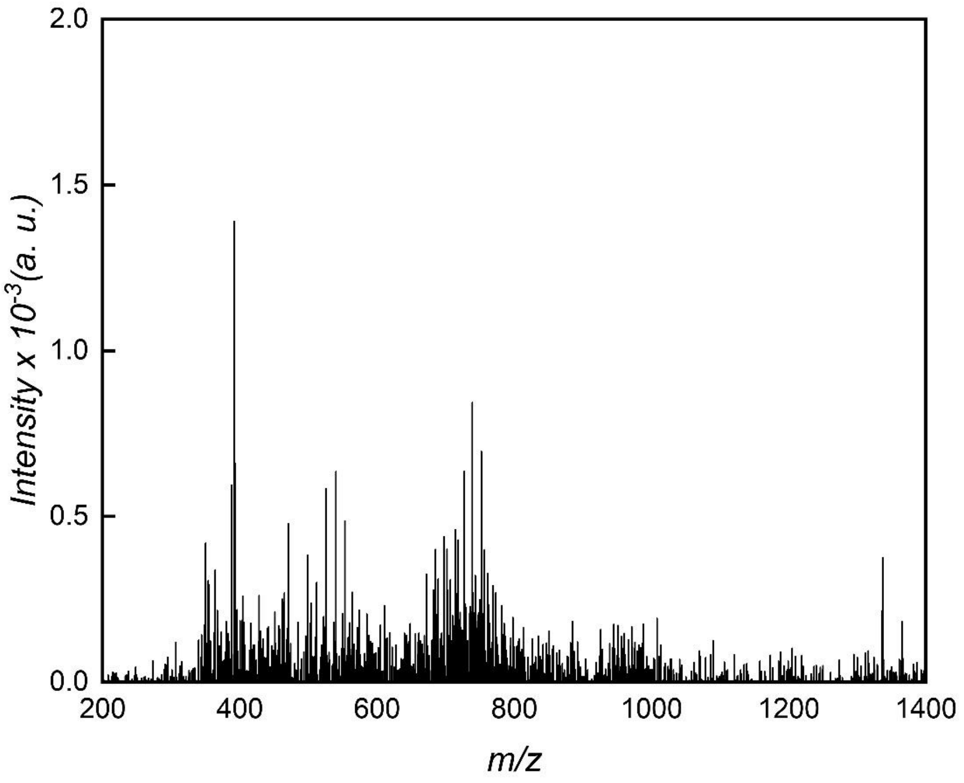

Figure S9. ESI mass spectrum of the sample MIM14BCD-NO3.

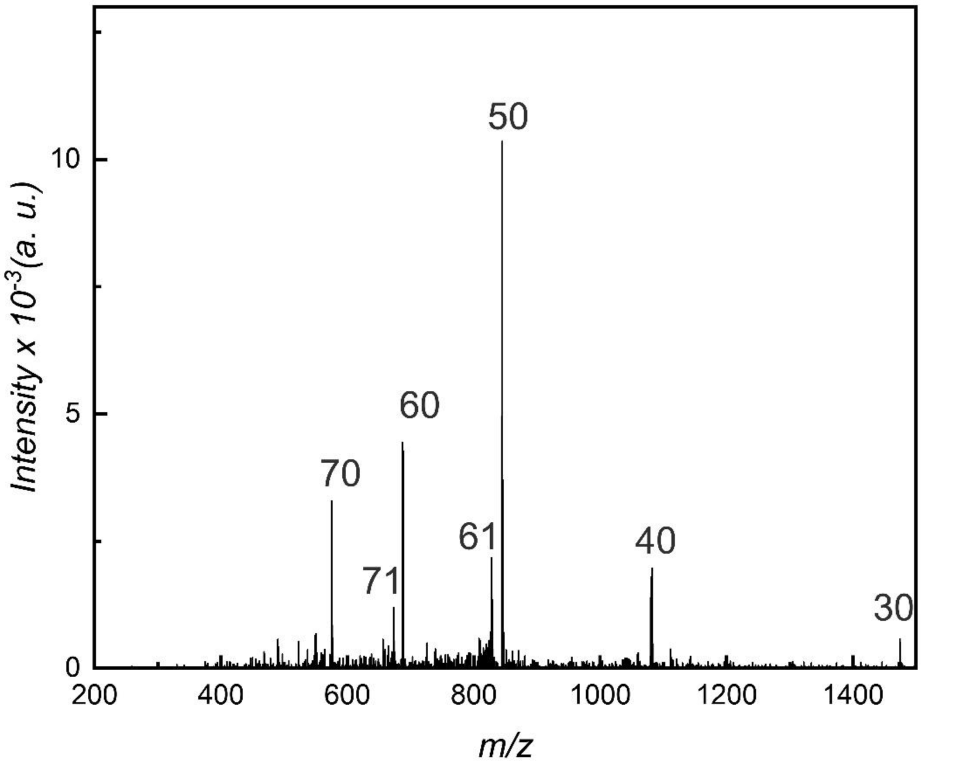

Figure S10. ESI mass spectrum of the sample MIM14BCD-ClO4. The first digit of the peaks' labels corresponds to the number of detached CLO_4^- and the second one to the number of attached OH^-.

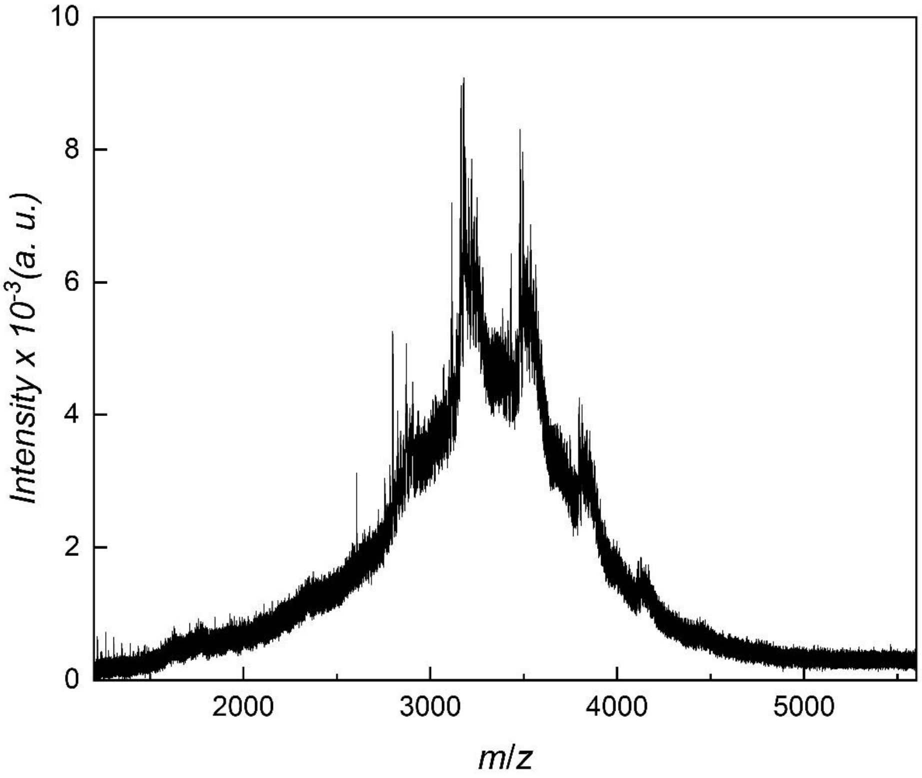

Figure S11. MALDI-TOF mass spectrum of the sample MIM7NBCD-Cl.

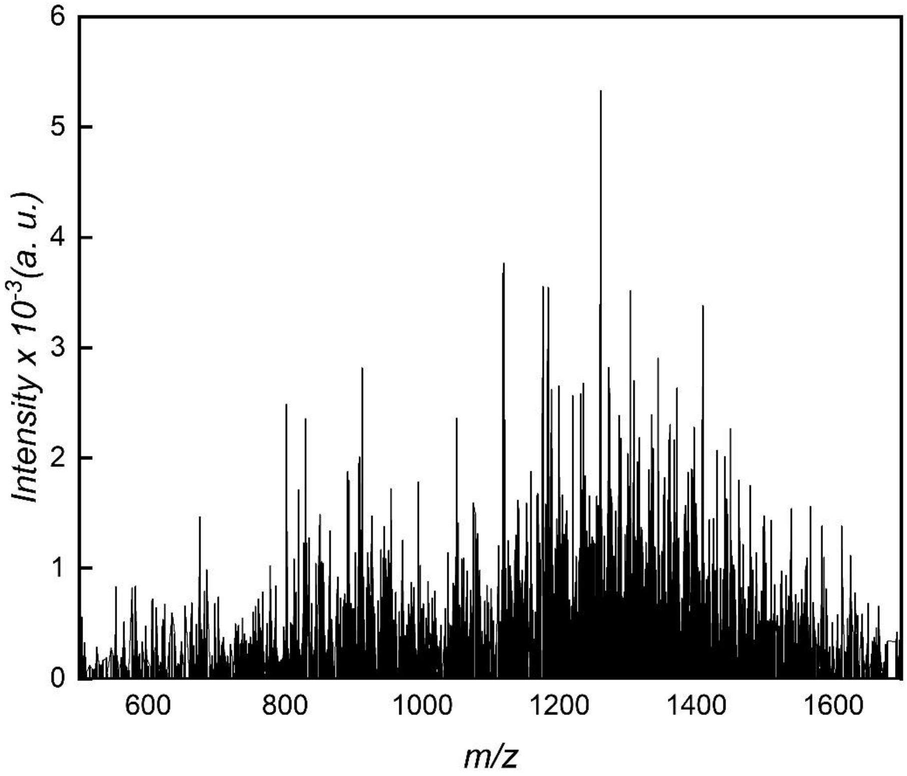

Figure S12. ESI mass spectrum of MIM7NBCD-TfO.

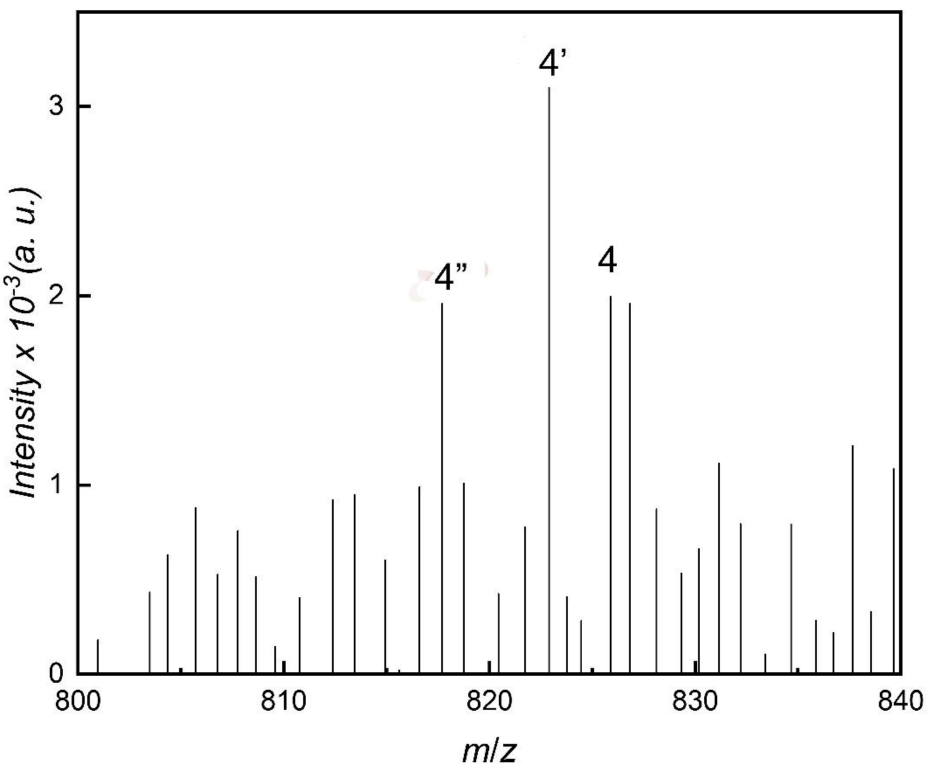

Figure S13. Expanded ESI mass spectrum of MIM7NBCD-Cl. The marked peaks correspond: **4''** to (MIM7NBCD-Cl - 4 Cl - 34)/4; **4'** to (MIM7NBCD-Cl - 4 Cl - 16)/4; **4** to (MIM7NBCD-Cl - 4 Cl)/4.

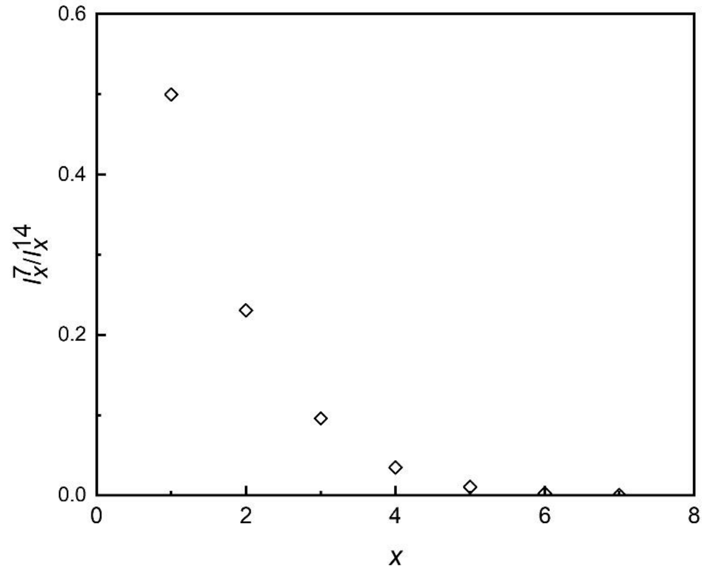

Figure S14. The ratio of MS intensity of analytes with 7 and 14 permanent charges from which *x* charges are free of counter-ion calculated from Equation 5 assuming 1-p ≈ 1.

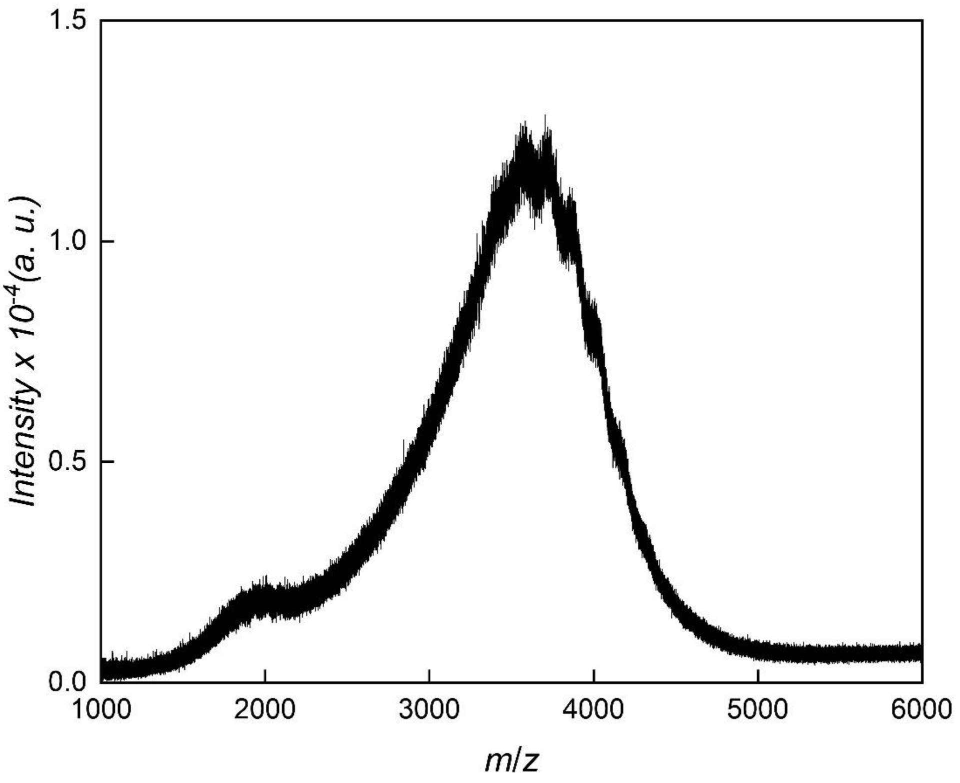

Figure S15. MALDI-TOF mass spectrum of sample MIM14BCD-Cl in the presence of TfOH at the molar ratio to Cl^- 1:1.

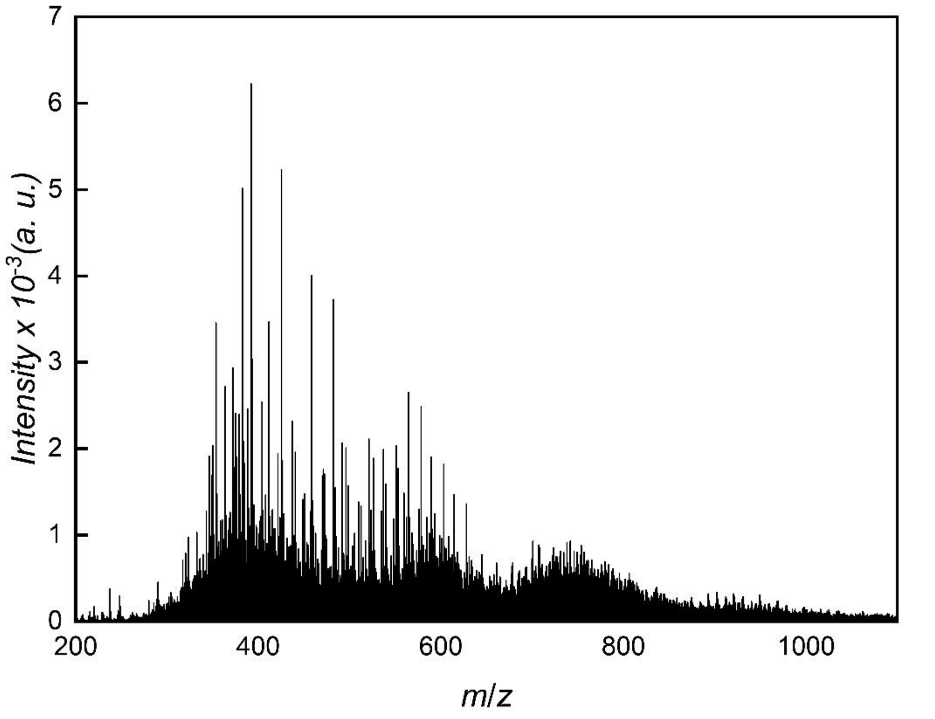

Figure S16. ESI mass spectrum of sample MIM14BCD-Cl in the presence of TfOH at the molar ratio to Cl^- 1:1.

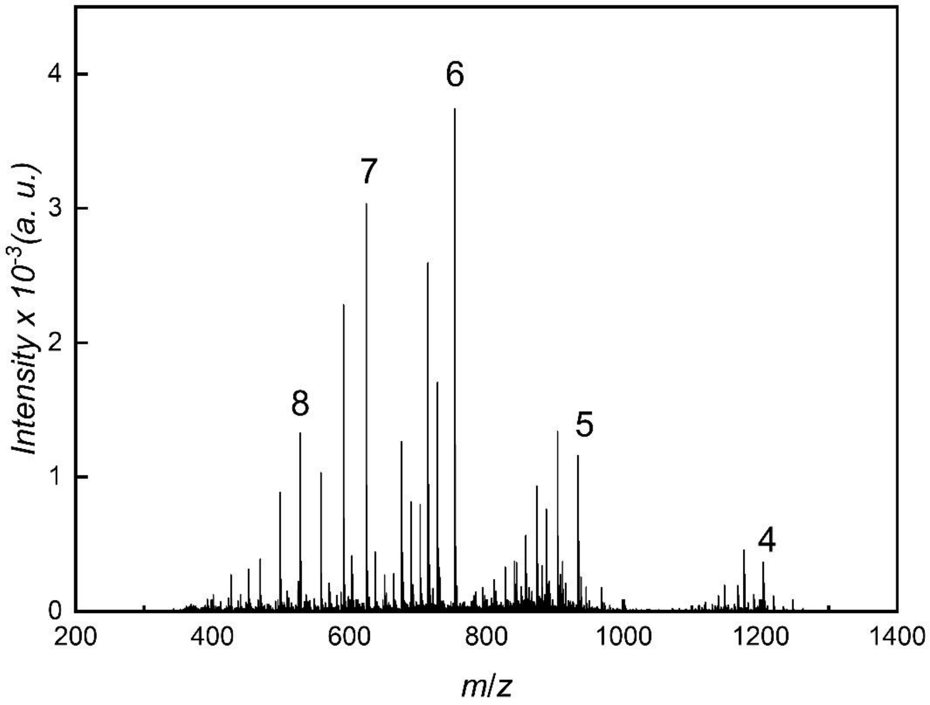

Figure S17. ESI mass spectrum of MIM14BCD-Cl in the presence of TfOH in 10x molar excess over Cl^-. The signals corresponding to adducts with TfO^- counter-ions only are denoted by the number of free charges.
